# Diagnostic sensitivity of RT-PCR assays on nasopharyngeal specimens for detection of SARS-CoV-2 infection: A Systematic Review and Meta-Analysis

**DOI:** 10.22088/cjim.13.0.139

**Published:** 2022

**Authors:** Marco Marando, Adriana Tamburello, Pietro Gianella, Rebecca Taylor, Enos Bernasconi, Tanja Fusi-Schmidhauser

**Affiliations:** 1 Internal Medicine Department, Ospedale Regionale di Lugano, Ente Ospedaliero Cantonale, Switzerland; 2 Division of Pneumology, Ospedale Regionale di Lugano, Ente Ospedaliero Cantonale, Switzerland; 3 Lancaster Medical School, Lancaster University, Lancaster, UK; 4 Division of Infectious Diseases, Ospedale Regionale di Lugano, Ente Ospedaliero Cantonale, Switzerland

**Keywords:** Nasopharyngeal swabs, SARS-CoV-2, RT-PCR assay, diagnostic yield

## Abstract

**Background:**

Reverse transcription polymerase chain reaction (RT-PCR) is the current standard of reference in the diagnosis of SARS-CoV-2 infection. In outpatient clinical practice, nasopharyngeal swab RT-PCR testing is still the most common procedure. The purpose of this systematic review and meta-analysis was to evaluate the sensitivity of RT-PCR nasopharyngeal assays.

**Methods:**

We searched three databases, including PubMed/MEDLINE, EMBASE, and Cochrane Library, using a comprehensive strategy. Studies investigating the sensitivity of SARS-CoV-2 RT-PCR nasopharyngeal assays in adults were included. Two reviewers extracted data and assessed trial quality independently. Pooled sensitivity and its confidence interval were computed using the meta package in R.

**Results:**

Thirteen studies were found eligible for the inclusion in the systematic review. Out of these, 25 different sub-studies were identified and included in the meta-analysis, which reported the sensitivities of 25 different nasopharyngeal RT-PCR assays. Finally, the overall pooled sensitivity resulted 89% (95% CI, 85.4 to 91.8%).

**Conclusion:**

Our study suggests that RT-PCR assays on nasopharyngeal specimens have a substantial sensitivity for diagnosing SARS-CoV-2 infection.

Since its appearance in December 2019, the severe acute respiratory syndrome coronavirus-2 (SARS-CoV-2) spread worldwide, being declared a pandemic in March 2020. As of August 14, 2021, there are globally 205,338,159 laboratory-confirmed cases and 4,333,094 confirmed deaths. The global mortality rate appears to be around 2.1 % (1). Even if the immunization drive is currently underway worldwide, early diagnosis of SARS-CoV-2 is still crucial to implement preventive medicine measures such as isolation, contact tracing and quarantines for close contacts of infected patients. Viral nucleic acid detection using reverse transcription polymerase chain reaction (RT-PCR) is considered the gold standard and the best single test to diagnose SARS-CoV-2 infection (2). Nasopharyngeal sampling remains the preferred route for SARS-CoV-2 diagnosis in outpatient practice and nasopharyngeal swabs are reported to be more sensitive than the oropharyngeal ones, even though only ~120 cases were included and results warranted further research (3). Available data indicate a wide range of sensitivity for RT-PCR assays, regardless of sampling location, although pooled sensitivities in meta-analysis show overall acceptable sensitivities (3-6). The aim of this systematic review and meta-analysis was to evaluate the sensitivity of RT-PCR assays specifically on nasopharyngeal specimens in a population of adults with a clinical suspicion of Coronavirus Disease – 2019 (COVID-19).

## Methods

This systematic review and meta-analysis was conducted according to the “Preferred Reporting Items for Systematic Reviews and Meta-Analyses” (PRISMA) statement, which represents an internationally recognized reporting guideline (7). Moreover, further recommendations on how to draft meta-analyses of diagnostic accuracy studies were followed (8).

**Search strategy :** On January 05, 2021 we conducted a comprehensive literature search on PubMed/MEDLINE, EMBASE, and Cochrane Library databases, in order to find appropriate published articles from December 1, 2019 on the diagnostic performance of reverse transcription polymerase chain reaction (RT-PCR) performed on first collected nasopharyngeal assays in SARS-CoV-2 infection in adults. The search algorithm combined these terms: ((COVID-19) OR (SARS-CoV-2)) AND ((RT-PCR) OR (real-time PCR) OR (real-time reverse transcription)) AND (sensitivity). Non-empirical research was excluded. We did not apply any language restriction. To perform the most accurate search possible, references of the retrieved articles were screened for additional entries.

**Study selection:** All studies or study subsets investigating the sensitivity for the diagnosis of SARS-CoV-2 RT-PCR assays on nasopharyngeal specimens in adults were deemed eligible for inclusion. The following exclusion criteria were applied: a) papers describing non-empirical research such as review articles, editorials or letters, comments, conference proceedings and case reports; b) case series with less than five patients; c) papers where the full text was in English, German, French, Italian or Spanish were not available. For the meta-analysis, studies with insufficient data were excluded. Two of the authors (AT and MM) performed an independent review of the retrieved titles and abstracts and then independently reviewed the full-text version in order to make a final decision. Finally, disagreements were resolved in a consensus meeting. 

**Data extraction:** We collected the following study information: study details (authors, date of publication, country, study design), sample size and specimen characteristics (gene(s) targeted whenever available, index test name, gold standard). Epidemiologic characteristics of patients and time interval from symptoms’ onset to specimen collection were not gathered due to their absence in most of the studies assessing the sensitivity. If a single study presented sensitivity results of different RT-PCR assays, we considered the study as many times as the number of the different assays analyzed. Thus, we included 25 different sub-studies in the quantitative analysis aiming to calculate the sensitivity of 25 different RT-PCR assays. 

**Bias assessment:** Risk of bias in each study was independently assessed by two of the reviewers (AT and MM) using the Diagnostic Precision Study Quality Assessment Tool (QUADAS-2), as recommended by the Cochrane Collaboration (9). Risk-of-bias plots were created using the robvis tool (10).

**Statistical analyses:** SARS-CoV-2 infection was defined by the positivity of a RT-PCR assay or as a result of a latent class analysis strategy, depending on the concerned study. Sensitivity of RT-PCR assays on nasopharyngeal specimens to diagnose SARS-CoV-2 infection was calculated, if not expressly indicated. Sensitivity was defined by the ratio between the number of patients tested positive for SARS-CoV-2 (A) and the sum of A with the number of those tested falsely negative altogether (B), according to the equation: sensitivity = (A)/(A + B). Individual study sensitivities and their standard errors were explored and visualized using a forest plot; five outlying studies (four reporting sensitivities greater than 99% and one indicating a particularly low percentage of 61.2%) were removed before calculating summary statistics on the remaining 20 studies. Pooled sensitivity and its confidence interval were computed by the mean of a univariate random effects model (meta package in R) (11, 12). The Clopper-Pearson method was used to create confidence intervals and the I2 statistic computed to determine heterogeneity. We applied a funnel plot to check for publication bias with contours depicting significance at the 10%, 5% and 1% levels, based on an assumption that a non-discriminatory (null) test would have a sensitivity of 50%. A trim-and-fill method was used to check for possible sources of bias. 

## Results

**Figure 1:**
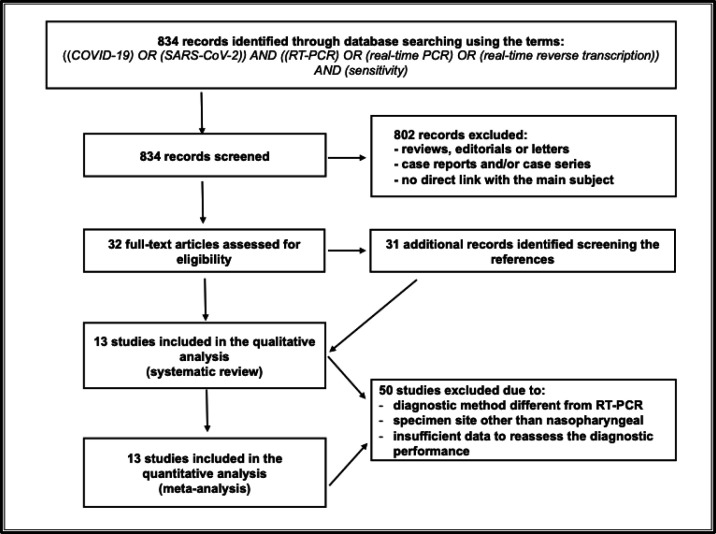
PRISMA Flow Diagram

**Literature search:** Our preliminary comprehensive literature search resulted in finding 834 articles. Thirty-one additional papers were retrieved through reference screening. After reviewing, 802 articles were excluded and 50 more articles did not meet the inclusion criteria after full-text assessment for eligibility. Figure 1 shows the study selection process. All thirteen articles included in the qualitative analysis (systematic review) were included in the quantitative analysis (meta-analysis), owing to their data completeness.

**Qualitative analysis (systematic review)**

In qualitative analysis we included thirteen full-text articles published over April 2020, comprising 8420 patients with laboratory-confirmed SARS-CoV-2 positivity. An overview of the study characteristics is presented in Table 1. Seven articles were prospective (53.8%) and six were retrospective (46.2%). Studies were conducted worldwide and data were available from the United States, Canada, Europe, Middle East, China and South America. Sample sizes ranged from 48 to 34,348 participants. Furthermore, out of these thirteen articles, we identified 25 different sub-studies, in order to analyze the sensitivity of the 25 different used RT-PCR assays. Detection rates are depicted in Table 2.

**Table 1:** Study characteristics

**First Author, year**	**Country**	**Month of publication**	**Study design**	**N° patients**	**Gene(s) targeted**	**Index test name**	**Reference standard**
Bisoffi.[13]	Italy	September	Prospective	346	S, RdRp	RQ-130	Latent class analysis (LCA)
Bisoffi.[13]	Italy	September	Prospective	346	N1, N2	CDC	Latent class analysis (LCA)
Bisoffi.[13]	Italy	September	Prospective	346	E + RdRp	In-house	Latent class analysis (LCA)
Bruce.[14]	U.S.	October	Retrospective	150	NA	NA	RT-PCR
Dugdale.[15]	U.S.	August	Retrospective	2736	NA	NA	RT-PCR
Fournier.[16]	France	October	Prospective	534	N	VitaPCR	RT-PCR
Freire-Paspuel.[17]	Ecuador	November	Prospective	48	E, RdRp	AccuPower	RT-PCR
Hasan.[18]	Qatar	July	Retrospective	132	NA	NA	RT-PCR
Jamal.[19]	Canada	June	Prospective	91	RdRp, E, N	Allplex	RT-PCR
Li.[20]	China	March	Retrospective	301	NA	NA	RT-PCR
Pavez.[21]	Chile	September	Retrospective	80	NA	SARS-CoV-2 RdRp plus EAV	RT-PCR
Pavez.[21]	Chile	September	Retrospective	80	NA	Real time fluorescent RT-PCR kit	RT-PCR
Pavez.[21]	Chile	September	Retrospective	80	NA	Detection kit for 2019-nCoV RNA	RT-PCR
Ridgway.[22]	U.S.	July	Prospective	34348	NA	Cepheid Xpert Xpress, Roche cobas, Abbott Id Now, BD Reagents, CDC, LabCorp, Quest, DiaSorin	RT-PCR
Ridgway.[22]	U.S.	July	Prospective	2443	NA	Cepheid Xpert Xpress, Roche cobas, Abbott Id Now, BD Reagents, CDC, LabCorp, Quest, DiaSorin	RT-PCR
Shen.[23]	China	September	Prospective	189	ORF1ab, N, RNP	NA	RT-PCR
Sutjipto.[24]	Singapore	August	Prospective	105	NA	A*Fortitude	RT-PCR
Zhen.[25]	U.S.	April	Retrospective	104	N1,N2, RP	Modified CDC	RT-PCR
Zhen.[25]	U.S.	April	Retrospective	104	S, ORF1ab	DiaSorin Molecular	RT-PCR
Zhen.[25]	U.S.	April	Retrospective	104	N	GenMark ePlex	RT-PCR
Zhen.[25]	U.S.	April	Retrospective	104	ORF1ab	Hologic Panther Fusion	RT-PCR

NA: not available.

**Table 2:** Sensitivity on nasopharyngeal specimens

**Authors**	**True positives / Detection rate of RT-PCR %**	**False negatives at RT-PCR**
Bisoffi.[13]	78 (91.2%)	7 (8.8%)
Bisoffi.[13]	64 (75.3%)	21 (24.7%)
Bisoffi.[13]	52 (61.2%)	33 (38.8%)
Bruce.[14]	138 (92%)	12 (8%)
Bruce.[14]	126 (84%)	24 (16%)
Dugdale.[15]	751 (95.4%)	36 (4.6%)
Fournier.[16]	155 (99.3%)	1 (0.7%)
Freire-Paspuel.[17]	30 (78.9%)	8 (21.1%)
Hasan.[18]	18 (95%)	1 (5%)
Jamal.[19]	64 (89%)	8 (11%)
Li.[20]	226 (75%)	75 (25%)
Pavez.[21]	73 (91%)	7 (9%)
Pavez.[21]	74 (93%)	6 (7%)
Pavez.[21]	77 (96%)	3 (4%)
Ridgway.[22]	4037 (81.7%)	906 (18.3%)
Ridgway.[22]	437 (96.7%)	15 (3.3%)
Shen.[23]	130 (91.5%)	12 (8.5%)
Shen.[23]	116 (81.7%)	26 (18.3%)
Shen.[23]	114 (80.3%)	28 (19.7%)
Shen.[23]	129 (90.8%)	23 (9.2%)
Sutjipto.[24]	62 (85%)	11 (15%)
Zhen.[25]	51 (100%)	0 (0%)
Zhen.[25]	51 (100%)	0 (0%)
Zhen.[25]	49 (96.1%)	2 (3.9%)
Zhen.[25]	51 (100%)	0 (0%)

**Quantitative analysis (meta-analysis)**

**Figure 2:**
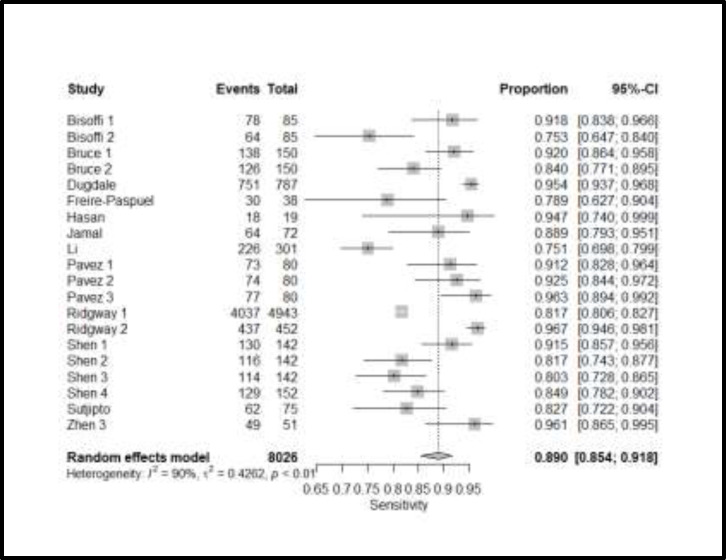
Meta-analysis results

After removing five outlying sub-studies, pooled sensitivity of RT-PCR assays on nasopharyngeal specimens was found to be 89% (95 % CI, 85.4 to 91.8%). Individual study sensitivities for the included studies are shown in the forest plot in Figure 2. 

**Figure 3:**
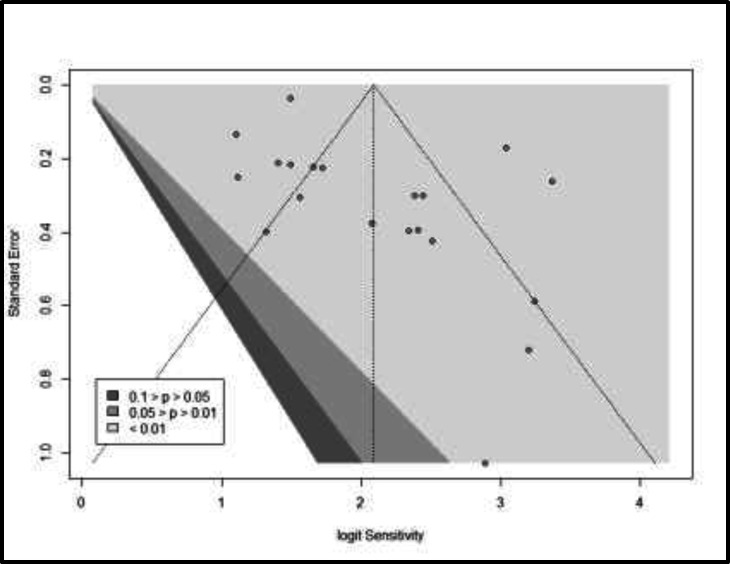
Funnel plot of the meta-analysis

Substantial heterogeneity is observed, with I2=89.9%, even after the removal of the outlying sub-studies. Figure 3 shows the funnel plot for the 20 included studies along with contours for 10%, 5% and 1% significance. Clearly these studies all show highly significant results, as they all sit widely to the right of the 99% significance contour, although visual inspection shows that they are not perfectly symmetrical about the pooled estimate. 

**Figure 4:**
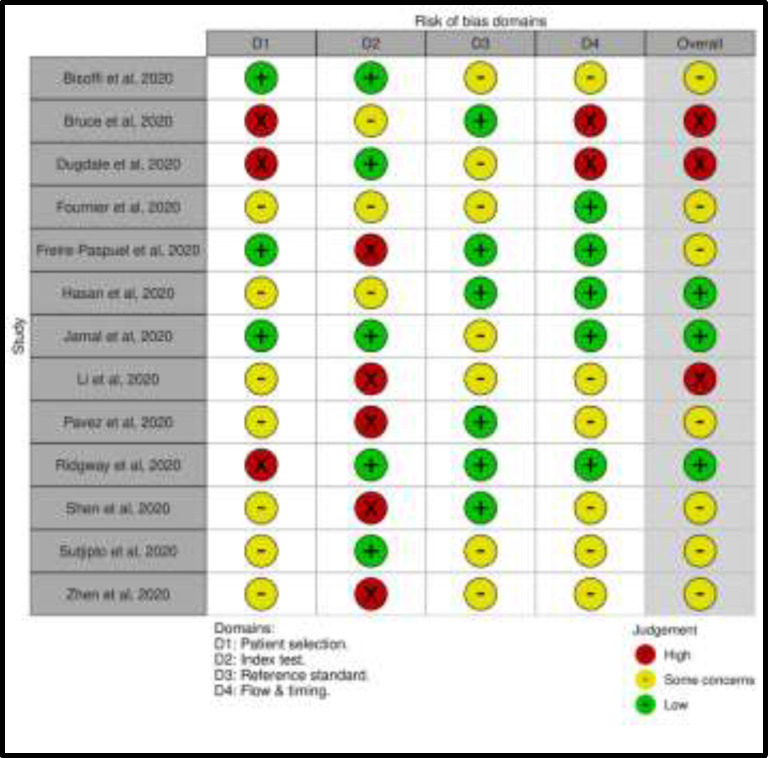
Methodological quality of the studies (individual assessment)

**Figure 5:**
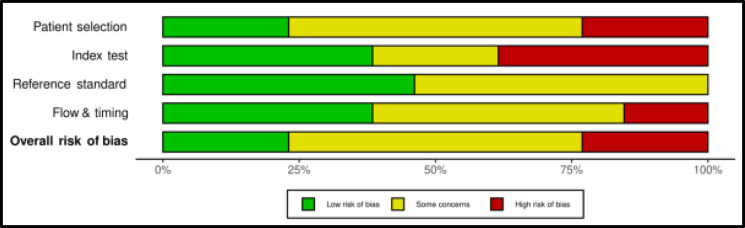
Summary of the methodological quality of the studies

**Quality assessment:** Risk-of-bias was rated as being moderate according to QUADAS-2. The most critical domains were patient selection, unclear in 7 studies (54%) and with a high risk of bias in 3 studies (23%) and index test, unclear in 3 studies (23%) and with a high risk of bias in 5 studies (38%). On the other hand, reference standard as well as flow and timing domains resulted in an overall lower risk of biases (Figure 4 and 5). All the included patients matched the review question and are thus likely to be diagnosed with the evaluative tests. 

## Discussion

This systematic review and meta-analysis suggests that RT-PCR assays on nasopharyngeal specimens achieve a pooled sensitivity of 89% (95 % CI, 85.4 to 91.8%) for diagnosing SARS-CoV-2 infection. Despite recent studies highlighted a comparable diagnostic accuracy of alternative specimens, such as salivary RT-PCR tests, nasopharyngeal swabs still maintain a critical role in the diagnostic workup of suspected COVID-19 (25, 26). In effect, although promising for its practicability and lower costs, saliva sampling is generally limited by the general spitting technique, which resulted in a significantly lower sensitivity for saliva than for nasopharyngeal swabs (27). 

Previous published work focused on comparing diagnostic accuracy of different available methods, such as RT-PCR assays on various specimens or radiological imaging. Specifically, a meta-analysis of Wikramaratna et al (3) published in early 2020 reported a test sensitivity of RT-PCR on nasopharyngeal specimens ranging from 96.40% (95% CI: 90.98 to 98.6) on symptom onset to 75.47% (95% CI: 66.88 to 82.51) on day 10 since symptom onset. In January 2021, Boger et al (28) reported a fairy good sensitivity for sputum (97.2%, 95% CI 90.3%-99.7%), while nasopharyngel/throat swabs and saliva demonstrated respectively a moderate sensitivity [(73.3%, 95% CI 68.1%-78.0%) and (62.3%, 95% CI 54.5%-69.6%)]. CT scan at best demonstrated a sensitivity of 87% (95% CI 85–90%) (29). The sensitivity of RT-PCR assays on nasopharyngeal specimens reported by these papers is consistent with our results, which considered trials published over a wide time span. Earlier testing generally results in a better sensitivity (30). We could not assess this issue in our study owing to the overall lack of information about the symptom onset in the included studies.

It should be noted that proper samples collection is of paramount importance to confer the optimal test accuracy. Thus, healthcare providers must be well-trained to ensure reliable results. Technically, nasopharyngeal swabs must be inserted horizontally, parallel to the palate, until no further insertion is possible; the swab must be twisted and left for a couple of seconds to significantly absorb the fluids (31). Our results highlight the fair sensitivity of nasopharyngeal specimens, even if false negative results (i.e. type 2 errors) may occur. To achieve a better accuracy, we assume that test repetition and the integration of RT-PCR assay results with epidemiological, clinical and radiological characteristics (i.e. pre-test probability) is essential to achieve an accurate diagnosis. Robust evidence on the relationship between sensitivity of RT-PCR assays on nasopharyngeal specimens and days from symptoms’ onset may further improve clinical guidance in daily practice. 

Nevertheless, our study has some limitations. Although we focused on the sensitivity of RT-PCR assays on nasopharyngeal specimens alone, the heterogeneity between included studies was still high. This may be explained by a “small study effect” and by different genes identified by the RT-PCR technique. An underlying publication bias that excluded studies with a sensitivity of nasopharyngeal specimens less than 50% seemed unlikely. Furthermore, it should be considered that as products of different companies have diverse detection thresholds, different clinical sensitivity for each test could result. Nonetheless, as compared with previous published meta-analysis on sensitivity of diagnostic methods for SARS-CoV-2, this study showed a similar or even smaller heterogeneity (3, 25, 26). Selection, recall and information biases related to a retrospective design of six out of 13 included studies need to be considered, even if a retrospective study design is justified by the current global health emergency. Furthermore, pre-analytic biases due to faults or difficulties in sampling (e.g. difficult sampling in uncooperative patients) or the conservation and transportation of samples and analytic biases such as differences in performance between various RT-PCR assays need to be acknowledged.

In conclusions our study suggests that RT-PCR assays for SARS-CoV-2 on nasopharyngeal specimens have a substantial sensitivity of 89% (95% CI, 85.4 to 91.8%). As a consequence of the sub-perfect sensitivity of nasopharyngeal swabs, we suggest that repetition of the RT-PCR test and further integration of molecular test results (i.e. PCR tests) with epidemiological, clinical and radiological characteristics (i.e. pre-test probability) is essential to achieve the most accurate possible diagnosis 
